# Association of Blood Pressure With Cause-Specific Mortality in Mexican Adults

**DOI:** 10.1001/jamanetworkopen.2020.18141

**Published:** 2020-09-25

**Authors:** Roberto Tapia-Conyer, Jesus Alegre-Díaz, Louisa Gnatiuc, Rachel Wade, Raúl Ramirez-Reyes, William G. Herrington, Sarah Lewington, Robert Clarke, Rory Collins, Richard Peto, Pablo Kuri-Morales, Jonathan Emberson

**Affiliations:** 1 Faculty of Medicine, National Autonomous University of Mexico, Mexico City, Mexico; 2 Clinical Trial Service Unit and Epidemiological Studies Unit, Nuffield Department of Population Health, University of Oxford, Oxford, United Kingdom; 3 Medical Research Council Population Health Research Unit, Nuffield Department of Population Health, University of Oxford, Oxford, United Kingdom

## Abstract

**Importance:**

Elevated blood pressure is a major cause of premature death, but there is little direct evidence demonstrating this association in studies of Hispanic populations.

**Objective:**

To assess the association between blood pressure and cause-specific mortality in a large cohort of Mexican adults with a high prevalence of uncontrolled diabetes.

**Design, Setting, and Participants:**

A total of 159 755 adults aged 35 years or older from 2 districts in Mexico City were recruited to this cohort study between April 1998 and September 2004 and followed up until January 2018. The present analyses focused on 133 613 participants who were aged 35 to 74 years and had no history of chronic disease besides diabetes.

**Exposure:**

Blood pressure.

**Main Outcomes and Measures:**

Cox regression, adjusted for confounders, yielded mortality rate ratios (RRs) for deaths of participants occurring between ages 35 and 74 years.

**Results:**

Of the 133 613 participants (43 263 [32.4%] men; mean [SD] age, 50 [11] years), 16 911 (12.7%) had self-reported previously diagnosed diabetes (including 8435 [6.3%] with uncontrolled diabetes, defined as hemoglobin A_1c_ ≥9%) and 6548 (4.9%) had undiagnosed diabetes. Systolic blood pressure (SBP) was associated with vascular mortality between ages 35 to 74 years, with each 20 mm Hg lower usual SBP associated with 35% lower vascular mortality (RR, 0.65; 95% CI, 0.61-0.68), including 48% lower stroke mortality (RR, 0.52; 95% CI, 0.47-0.59) and 32% lower ischemic heart disease mortality (RR, 0.68; 95% CI, 0.63-0.74). These RRs were broadly similar in those with and without diabetes. Compared with those without diabetes and SBP less than 135 mm Hg at recruitment, the vascular mortality RR was 2.8 (95% CI, 2.4-3.3) for those without diabetes and SBP of 155 mm Hg or greater, 4.7 (95% CI, 4.1-5.4) for those with uncontrolled diabetes and SBP less than 135 mm Hg, and 8.9 (95% CI, 7.2-11.1) for those with uncontrolled diabetes and SBP of 155 mm Hg or greater. Lower SBP was also associated with decreased kidney-related mortality (RR per 20 mm Hg lower usual SBP, 0.69; 95% CI, 0.64-0.74), decreased mortality from infection (RR, 0.81; 95% CI, 0.71-0.91), and decreased mortality from hepatobiliary disease (RR, 0.87; 95% CI, 0.78-0.98), but not decreased neoplastic or respiratory mortality. SBP was more informative for vascular mortality than other blood pressure measures (eg, compared with SBP, diastolic blood pressure was only two-thirds as informative).

**Conclusions and Relevance:**

Blood pressure was most strongly associated with vascular and kidney-related mortality in this Mexican population, with particularly high absolute excess mortality rates among individuals with diabetes. The findings reinforce the need for more widespread use of blood pressure–lowering medication in Mexico, particularly among those with diabetes.

## Key Points

**Question:**

What is the association between blood pressure and mortality in Mexican adults and how does it vary with other characteristics such as diabetes?

**Findings:**

In this cohort study of 133 613 Mexican adults aged 35 to 74 years with high levels of adiposity and uncontrolled diabetes, blood pressure was most strongly associated with death from vascular and kidney disease, and the absolute excess mortality rates associated with elevated blood pressure were much higher in individuals with diabetes.

**Meaning:**

The results reinforce the need for more widespread use of blood pressure–lowering medication in Mexico, particularly for individuals with diabetes.

## Teaser

This cohort study of Mexican adults aged 35 to 74 years examines the association of elevated blood pressure with excess mortality rates, particularly for individuals with diabetes.

## Introduction

Elevated blood pressure is a major cause of death and disability. Using evidence from 2 large meta-analyses of prospective studies^1,2^ and 1 large health-record linkage study in the UK,^3^ the 2017 Global Burden of Disease Collaborators estimated that elevated systolic blood pressure (SBP), defined as SBP higher than 110 to 115 mm Hg, was the leading cause of disability-adjusted life-years lost among women, and the second-leading cause among men (behind only tobacco use).^4^ In a 2002 meta-analysis of data from 61 cohorts involving 1 million people,^1^ each 20 mm Hg lower SBP was associated with at least a halving in the death rates from ischemic heart disease, stroke, and other vascular causes between ages 40 and 69 years. Since most of these studies were completed, however, vascular mortality has decreased in many countries while obesity (a key determinant of blood pressure) and diabetes have increased.^5,6^ Moreover, most previous prospective studies have been conducted in high-income countries, so the extent to which their findings are generalizable to other countries with different health care systems and risk factor distributions is unclear.

We previously found that, among 150 000 Mexican adults recruited into a prospective study between 1998 and 2004, diabetes was more strongly associated with mortality than was expected based on studies done in other populations.^7,8^ Using updated data from the Mexico City Prospective Study, the main aim of this study was to assess the association between blood pressure and 16-year cause-specific mortality in this Mexican population and in particular to examine how the association differed among those with and without diabetes.

## Methods

### Recruitment and Baseline Assessment

From 1998 to 2004, households within 2 districts of Mexico City (Coyoacán and Iztapalapa) were visited systematically, and all residents aged 35 years or older were invited to join the study; 159 755 residents agreed to participate.^9^ Trained nurses recorded sociodemographic and lifestyle factors, current medications, and medical history. Seated SBP and diastolic blood pressure (DBP) were measured 3 times (at 1-minute intervals) to the nearest 1 mm Hg using a standard mercury sphygmomanometer. Weight, height, waist circumference, and hip circumference were measured, and a 10 mL blood sample was collected. Hemoglobin A_1c_ was measured from the buffy coat sample using validated high-performance liquid chromatography methods^10^ on HA-8180 analyzers (Arkray Inc) with calibrators traceable to International Federation of Clinical Chemistry and Laboratory Medicine standards.^11^ Ethics approval was granted by the Mexican Ministry of Health, Mexican National Council of Science and Technology, and the University of Oxford (United Kingdom). All participants provided written informed consent. This study followed the Strengthening the Reporting of Observational Studies in Epidemiology (STROBE) reporting guideline.

### Resurvey Assessment

From June 2015 to February 2019, a median (interquartile range [IQR]) follow-up length of 15.5 (14.3-18.3) years, 10 144 surviving participants (7100 [70.0%] women) were revisited in their homes and agreed to participate in a resurvey. During this visit, seated blood pressure was measured 3 times using an automated upper arm blood pressure monitor (A&D Medical).

### Mortality Follow-up

Mortality was tracked until January 2018 through probabilistic linkage to the national death register, which encodes all diseases listed on the death certificate according to the *International Statistical Classification of Diseases and Related Health Problems, Tenth Revision* (*ICD-10*).^12^ Study clinicians then reviewed and, if necessary, recoded the underlying cause of death (eg, accepting diabetes as the underlying cause only for deaths because of acute diabetic crises^7^). The single underlying cause of death was then used for prospective analyses (detailed in the following section). Vascular deaths were categorized as ischemic heart disease (IHD), stroke, and other vascular causes (eTable 1 in the Supplement). Nonvascular causes of death were classified as neoplastic, kidney, infective, hepatobiliary, respiratory, and other causes of death.

### Statistical Analysis

All prospective analyses excluded participants with (self-reported) prior diagnosis of angina, heart attack, stroke, cancer, chronic kidney disease (CKD), cirrhosis, or emphysema to reduce the risk of reverse causality bias (ie, prior diseases may alter the level of blood pressure prior to enrollment). Participants who had missing or extreme values of any covariate data were also excluded. Remaining participants were then classified into 6 baseline SBP categories (<125, ≥125 to <135, ≥135 to <145, ≥145 to <155, ≥155 to <165, and ≥165 mm Hg). The main analyses were limited to participants aged 35 to 74 years at recruitment and to deaths occurring before age 75 years. However, additional supplementary analyses are provided for those aged 75 to 84 years (and deaths at ages 75 to 84 years) (eAppendix in the Supplement).

Cox regression was used to estimate the associations between SBP (in the above 6 groups) and particular causes of death. Analyses were adjusted for age-at-risk, sex, location (2 districts), education (ie, university or college, high school, elementary school, or other), smoking (ie, never, former, occasional, <10 cigarettes per day, ≥10 cigarettes per day), alcohol intake (ie, none, former use, current use), leisure-time physical activity (ie, none, up to twice weekly, at least 3 times weekly), anthropometry (ie, height, weight, waist, and hip, each split into 4 equally sized groups), and diabetes status (divided into 5 groups: no diabetes; undiagnosed diabetes [ie, no previous diagnosis but hemoglobin A_1c_ ≥6.5%; to convert to proportion of total hemoglobin, multiply by 0.01]; previously diagnosed diabetes with hemoglobin A_1c_ <9%; previously diagnosed diabetes with hemoglobin A_1c_ ≥9% to <11%; and previously diagnosed diabetes with hemoglobin A_1c_ ≥11%).^8^ These confounders were specified a priori based on their known associations with blood pressure and with vascular mortality in other populations. In figures, mortality rate ratios (RRs; estimated using the Cox hazard ratios) for each group are shown together with group-specific 95% CIs (including for the reference group with RR of 1.0).^13^ Separate RRs at levels of each confounder were estimated by inclusion of the relevant interaction terms in the regression models (while holding other confounders constant).

To assess the strength of the association of SBP with cause-specific mortality, baseline SBP was then included in the model as a continuous variable, with RRs estimated for each 20 mm Hg lower SBP. However, because such analyses will underestimate the association of long-term “usual” blood pressure with risk because of regression dilution bias,^14^ we divided the log RR (and its standard error) by the regression dilution ratio for SBP, which was conservatively estimated to be 0.76) (eFigure 1 in the Supplement). Similarly, for the categorical analyses described earlier, the RRs were plotted against the expected mean usual level in each group (calculated as M + 0.76 × [b − M], where *b* is the group-specific mean baseline level and *M* is the overall mean).^15^

We then estimated the association between blood pressure and mortality separately for participant cohorts with and without previously diagnosed diabetes. For analyses of vascular mortality, we further separated those with diabetes according to glycemic control (hemoglobin A_1c_ <9% vs ≥9%), did separate analyses within each strata of the main confounders, and estimated the RRs separately for deaths occurring within 5 years, between 5 and 10 years, and 10 years or more after recruitment. Comparative analyses of DBP were also performed. Finally, to compare the strength of association of different blood pressure indices recorded at baseline with vascular mortality rates, the relative “informativeness” of a given blood pressure index (SBP, DBP, or some combination of them) was estimated from the age-adjusted and confounder-adjusted χ^2^ statistic relating it to cause-specific mortality.^1^

Analyses were performed with SAS version 9.4 (SAS Institute) and R version 3.5.3 (R Project for Statistical Computing). All *P* values are 2-sided and considered statistically significant at the .05 level.

## Results

### Included Participants

Of the 159 755 potential study participants, 8135 (5.1%) were excluded because of a prior history of chronic disease and a further 8883 (5.6%) were excluded because of missing or extreme values. Among the remaining participants, 133 613 (93.6%) were aged between 35 and 74 years at recruitment (43 263 [32.4%] men; mean [SD] age, 50 [11] years) (Table) and 7461 (5.2%) were aged between 75 and 84 years at recruitment (2789 [37.4%] men; mean [SD] age, 79 [3] years) (eTable 2 in the Supplement). Mean (SD) SBP in the men increased from 123 (12) mm Hg between ages 35 and 44 years to 136 (18) mm Hg between ages 75 and 84, while mean SBP in women increased from 119 (13) mm Hg between ages 35 and 44 years to 139 (19) mm Hg between ages 75 and 84 (eFigure 2 in the Supplement). DBP increased with age in both sexes up to approximately age 60, after which there was no further increase and a slight reduction in mean levels. Use of blood pressure–lowering medication increased from 2% of men (377 of 15 754) and 4% of women (1488 of 35 408) between ages 35 and 44 years to 26% of men (730 of 2789) and 39% of women (1831 of 4672) between ages 75 and 84 years. The characteristics of 9031 resurveyed participants without prior chronic disease and aged between 35 and 74 years at original study recruitment are provided in eTable 3 in the Supplement.

**Table.** Baseline Characteristics of 133 613 Participants Without Prior Chronic Disease (Other Than Diabetes) and Between Ages 35 and 74 at Recruitment^a^

Characteristic	Participants, No. (%)						
	Overall (N = 133 613)	Baseline systolic blood pressure, mm Hg					
		<125 (n = 68 234)	125-134 (n = 32 041)	135-144 (n = 17 967)	145-154 (n = 8177)	155-164 (n = 3902)	≥165 (n = 3292)
Age, mean (SD), y	50.4 (10.8)	47.0 (9.5)	51.5 (10.5)	54.6 (10.7)	57.1 (10.3)	58.6 (9.8)	59.9 (10.8)
Men	43 263 (32.4)	20 272 (29.7)	11 574 (36.1)	6381 (35.5)	2733 (33.4)	1286 (33.0)	1017 (30.9)
Blood pressure, mean (SD), mm Hg							
Systolic	127 (16)	115 (8)	130 (3)	139 (3)	149 (3)	159 (3)	178 (12)
Diastolic	83 (10)	77 (7)	85 (6)	90 (7)	94 (8)	97 (9)	104 (12)
Taking blood pressure–lowering medication	17 908 (13.4)	3306 (4.8)	4456 (13.9)	4166 (23.2)	2699 (33.0)	1533 (39.3)	1748 (53.1)
Socioeconomic status and lifestyle behaviors							
Resident of Coyoacán	52 547 (39.3)	25 190 (36.9)	13 104 (40.9)	7591 (42.2)	3397 (41.5)	1711 (43.8)	1554 (47.2)
University or college educated	21 474 (16.1)	13 333 (19.5)	4710 (14.7)	2103 (11.7)	762 (9.3)	321 (8.2)	245 (7.4)
Current smoking	43 869 (32.8)	24 411 (35.8)	10 254 (32.0)	5346 (29.8)	2145 (26.2)	984 (25.2)	729 (22.1)
Current alcohol use	99 915 (74.8)	51 272 (75.1)	24 166 (75.4)	13 391 (74.5)	5891 (72.0)	2828 (72.5)	2367 (71.9)
Any regular leisure-time physical activity	29 766 (22.3)	15 680 (23.0)	7167 (22.4)	3811 (21.2)	1719 (21.0)	782 (20.0)	607 (18.4)
Physical measurements, mean (SD)							
BMI	29.1 (4.9)	28.2 (4.5)	29.8 (5.0)	30.3 (5.3)	30.5 (5.4)	30.6 (5.5)	30.8 (5.6)
Circumference, cm							
Waist	94 (12)	91 (11)	96 (11)	98 (12)	98 (12)	99 (12)	100 (12)
Hip	105 (11)	103 (10)	106 (11)	107 (11)	107 (12)	107 (12)	108 (12)
Diabetes status and hemoglobin A_1C_							
No diabetes	110 154 (82.4)	60 244 (88.3)	25 911 (80.9)	13 545 (75.4)	5773 (70.6)	2610 (66.9)	2071 (62.9)
Undiagnosed diabetes	6548 (4.9)	2221 (3.3)	1793 (5.6)	1293 (7.2)	625 (7.6)	330 (8.5)	286 (8.7)
Diagnosed diabetes with hemoglobin A_1c_ <9%	8476 (6.3)	2694 (3.9)	2201 (6.9)	1619 (9.0)	923 (11.3)	532 (13.6)	507 (15.4)
Diagnosed diabetes with hemoglobin A1c ≥9% and <11%	4299 (3.2)	1473 (2.2)	1096 (3.4)	776 (4.3)	471 (5.8)	241 (6.2)	242 (7.4)
Diagnosed diabetes with hemoglobin A_1c_ ≥11%	4136 (3.1)	1602 (2.3)	1040 (3.2)	734 (4.1)	385 (4.7)	189 (4.8)	186 (5.7)
Medication use							
Any antithrombotic	3109 (2.3)	1241 (1.8)	774 (2.4)	515 (2.9)	304 (3.7)	137 (3.5)	138 (4.2)
Any lipid lowering	670 (0.5)	240 (0.4)	186 (0.6)	126 (0.7)	61 (0.7)	31 (0.8)	26 (0.8)

Abbreviation: BMI, body mass index (calculated as weight in kilograms divided by height in meters squared).

SI conversion factor: To convert hemoglobin A_1c_ to proportion of total hemoglobin, multiply by 0.01.

^a^ Table excludes individuals with prior chronic disease at recruitment (ie, ischemic heart disease, stroke, chronic kidney disease, cirrhosis, cancer, or emphysema), missing or out-of-range data on any analysis covariate (ie, sex, district of residence, educational level attained, anthropometry, smoking status, alcohol intake, leisure time physical activity, or diabetes status), uncertain follow-up, or missing or out-of-range blood pressure (defined as SBP <80 or >250 mm Hg, DBP <40 or >150 mm Hg, or pulse pressure <15 mm Hg).

### SBP and Vascular Mortality

**Figure 1.**
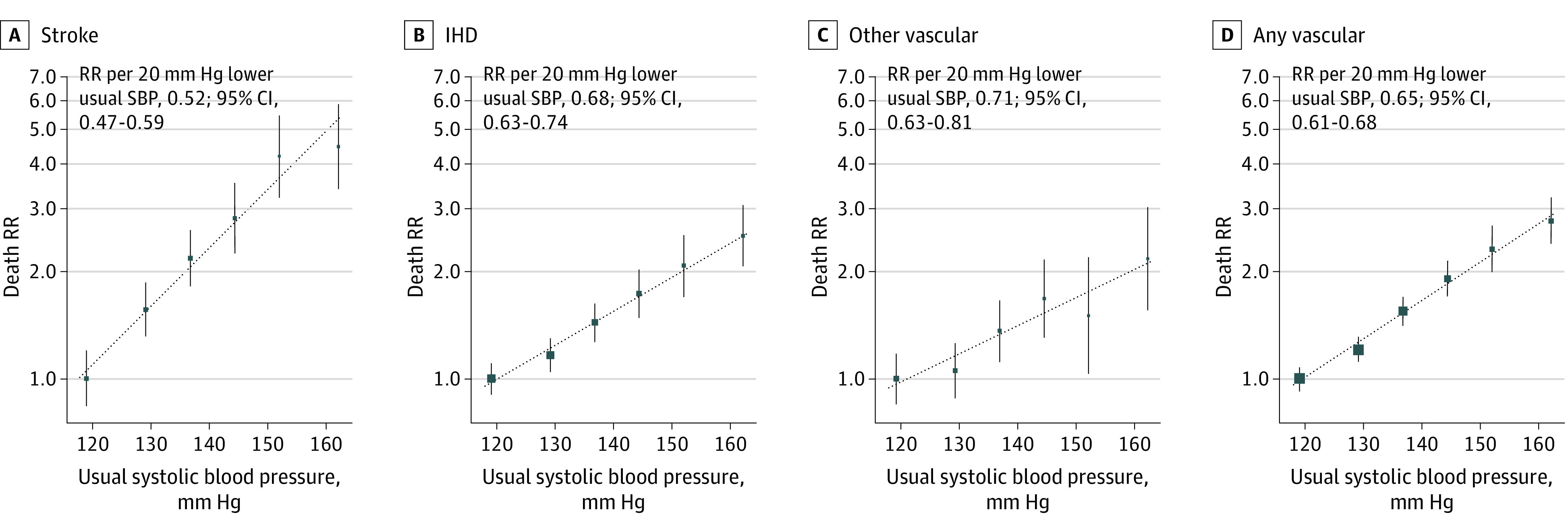
Association of Systolic Blood Pressure (SBP) With Cause-Specific Vascular Mortality Between Ages 35 and 74 Years Analyses excluded participants with prior chronic diseases (ie, ischemic heart disease [IHD], stroke, chronic kidney disease, cirrhosis, cancer, or emphysema) apart from diabetes. The mortality rate ratios (RRs) for the 6 baseline SBP categories were plotted against the expected long-term usual SBP level in each group. The error bars through each point represent group-specific 95% CIs, with the area of each square proportional to the amount of statistical information. Estimates of RR were stratified by age-at-risk (in 5-year ranges) and are adjusted for sex, district of residence, highest education level attained, smoking status, alcohol intake, leisure-time physical activity, measures of anthropometry, and diabetes status.

During a median (interquartile range) follow-up among survivors of 15.5 years (14.7-16.7), 2508 participants died from vascular causes between ages 35 and 74 years, including 574 (22.9%) from stroke, 1406 (56.1%) from IHD, and 528 (21.1%) from other vascular causes. For stroke, IHD, and the composite of all other vascular causes of death (and, hence, for the composite of all vascular deaths combined), the association of SBP with mortality was continuous and log-linear throughout the range studied (Figure 1). Across this age range, each 20 mm Hg lower usual SBP was associated with a 48% lower stroke mortality rate (RR, 0.52; 95% CI, 0.47-0.59), a 32% lower IHD mortality rate (RR, 0.68; 95% CI, 0.63-0.74), and a 29% lower mortality rate from other vascular causes (RR, 0.71; 95% CI, 0.63-0.81). When combined, each 20 mm Hg lower usual SBP was associated with a 35% lower rate of death from any vascular cause (RR, 0.65; 95% CI, 0.61-0.68).

### Age-Specific and Diabetes-Specific Associations

**Figure 2.**
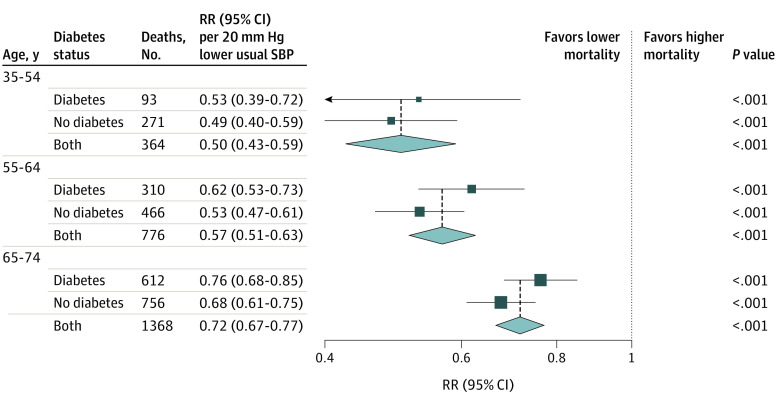
Association of Systolic Blood Pressure (SBP) With Vascular Mortality by Age and History of Previously Diagnosed Diabetes Analyses excluded participants with prior chronic diseases (ie, ischemic heart disease, stroke, chronic kidney disease, cirrhosis, cancer, or emphysema) apart from diabetes. Within each age-at-risk group, the mortality rate ratio (RR) estimates in those with and without previously diagnosed diabetes were stratified by age-at-risk (in 5-year ranges) and adjusted for sex, district of residence, highest education level attained, smoking status, alcohol intake, leisure-time physical activity, and measures of anthropometry. The overall RR estimates are then also adjusted for diabetes. A test for trend in the log RR across the 3 age-at-risk categories shown yielded a χ^2^ statistic of 21.5 (*P* < .001). Trends described in the heading of the point estimate line indicate lower and higher mortality associated with lower SBP.

The proportional reduction in the vascular mortality rate associated with a 20 mm Hg lower usual SBP was greater at younger than at older ages but, for a given age, was broadly similar in those with and without previously diagnosed diabetes (Figure 2). Each 20 mm Hg lower usual SBP was associated with 50% lower vascular mortality between ages 35 and 54 years (RR, 0.50; 95% CI, 0.43-0.59), 43% lower vascular mortality between ages 55 and 64 years (RR, 0.57; 95% CI, 0.51-0.63), and 28% lower vascular mortality between ages 65 and 74 (RR, 0.72; 95% CI, 0.67-0.77). eFigure 3 in the Supplement shows that the shape of the association between SBP and vascular mortality, and its components, remained continuous and log-linear at older ages.

**Figure 3.**
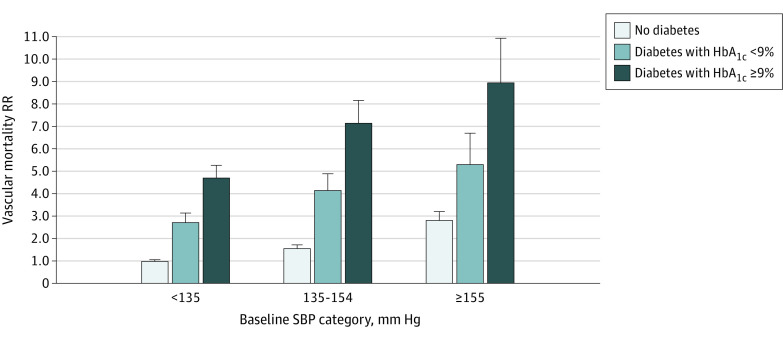
Absolute Excess Vascular Mortality Between Ages 35 and 74 Years Associated With Systolic Blood Pressure (SBP) by History and Control or Previously Diagnosed Diabetes Analyses excluded participants with prior chronic diseases (ie, ischemic heart disease, stroke, chronic kidney disease, cirrhosis, cancer, or emphysema) apart from diabetes. For the 9 groups shown, the mortality rate ratio (RR) estimates are stratified by age-at-risk (in 5-year ranges) and adjusted for sex, district of residence, highest education level attained, smoking status, alcohol intake, leisure-time physical activity, and measures of anthropometry. The error bar extending above each column extends to the upper 95% confidence limit of the RR. The mean usual SBP in the 3 SBP categories shown was 121, 139, and 158 mm Hg, respectively.

Because individuals with diabetes had much higher death rates than those without diabetes, the absolute excess vascular mortality associated with higher blood pressure was greater among those with diabetes (and especially among those with diabetes and poor glycemic control) (Figure 3). Classifying individuals by both baseline SBP and diabetes, compared with those without diabetes and SBP less than 135 mm Hg, the vascular mortality RR between ages 35 and 74 years was 2.8 (95% CI, 2.4-3.3) for those without diabetes and SBP of 155 mm Hg or greater, 4.7 (95% CI, 4.1-5.4) for those with uncontrolled diabetes and SBP less than 135 mm Hg, and 8.9 (95% CI, 7.2-11.1) for those with uncontrolled diabetes and SBP of 155 mm Hg or greater.

### Sensitivity Analyses

At any given age, the associations between SBP and vascular mortality were similar for men and women (eFigure 4 in the Supplement) and were also broadly similar irrespective of city district, smoking, drinking, physical activity, or markers of adiposity and diabetes (eFigure 5 in the Supplement). However, a significant trend toward stronger associations among those with higher levels of education was seen. When vascular deaths between ages 35 and 74 years were subdivided according to time since recruitment (ie, <5 years, 5 to <10 years, and 10 or more years), the RR per 20 mm Hg lower SBP was also similar in each period of follow-up (eFigure 6 in the Supplement). The main results were also almost identical when analyses were repeated after additional adjustment for antithrombotic and lipid-lowering medication.

### Informativeness of Different Blood Pressure Indices

Among the different blood pressure indices considered, including SBP, DBP, pulse pressure (ie, SBP minus DBP), mid–blood pressure (ie, [SBP + DBP]/2), and mean arterial pressure (ie, 2/3 SBP + 1/3 DBP), a single measurement of SBP was found to be the most statistically informative for stroke, IHD, and vascular mortality (eTable 4 in the Supplement). Compared with SBP, DBP was two-thirds as informative for stroke and vascular mortality, but only half as informative for IHD mortality. Each 10 mm Hg lower usual DBP was associated with 45% lower stroke mortality (RR, 0.55; 95% CI, 0.48-0.62), 26% lower IHD mortality (RR, 0.74; 95% CI, 0.68-0.80), and 32% lower mortality from any vascular cause (RR, 0.68; 95% CI, 0.64-0.73) between ages 35 between 74 years. Pulse pressure was less informative than the other indices.

### SBP and Nonvascular Mortality

**Figure 4.**
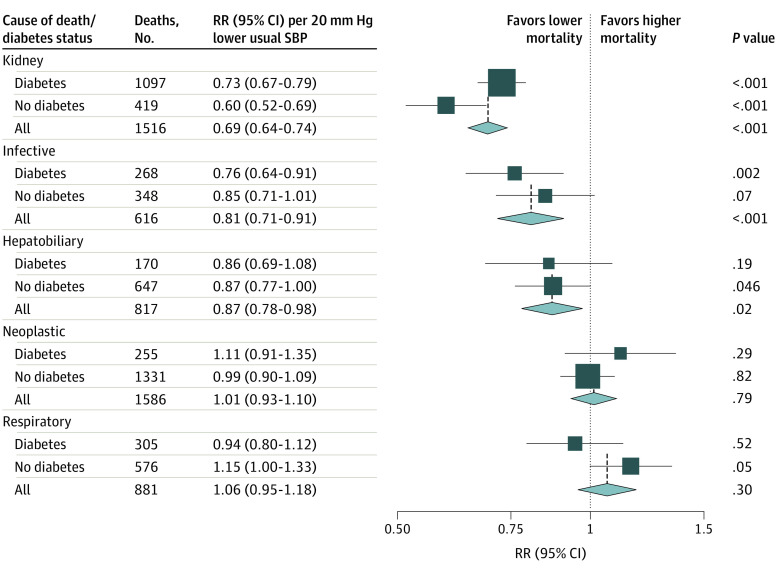
Association of Systolic Blood Pressure (SBP) With Nonvascular Mortality Between Ages 35 and 74 Years by History of Previously Diagnosed Diabetes Analyses excluded participants with prior chronic diseases (ie, ischemic heart disease, stroke, chronic kidney disease, cirrhosis, cancer, or emphysema) apart from diabetes. For each cause of death, the mortality rate ratio (RR) estimates in those with and without previously diagnosed diabetes are stratified by age-at-risk (in 5-year ranges) and adjusted for sex, district of residence, highest education level attained, smoking status, alcohol intake, leisure-time physical activity, and measures of anthropometry. The overall RR estimates are then also adjusted for diabetes. Trends described in the heading of the point estimate line indicate lower and higher mortality associated with lower SBP. In addition to the nonvascular deaths shown, there were an additional 359 deaths between ages 35 and 74 from an acute diabetic crisis and 662 deaths from other known or unknown causes. The association between SBP and mortality from these other causes is shown in eFigure 7 and eFigure 8 in the Supplement.

Figure 4 shows the strengths of association between SBP and major nonvascular causes of death, both overall and separately in those with and without previously diagnosed diabetes. (The shapes of these associations are shown in eFigure 7 and 8 in the Supplement.) Lower SBP was associated with lower risk of death from kidney disease (RR per 20 mm Hg lower usual SBP, 0.69; 95% CI, 0.64-0.74), particularly among those without diabetes, lower risk of death from infection (RR, 0.81; 95% CI, 0.71-0.91), and lower risk of death from hepatobiliary disease (RR, 0.87; 95% CI, 0.78-0.98). However, lower SBP was not associated with lower risk of neoplastic or respiratory death.

## Discussion

In this large study of Mexican adults, SBP was log-linearly associated with vascular mortality, with no threshold level below which a lower SBP was not associated with a lower risk, at least down to an SBP of approximately 120 mm Hg. For death at age younger than 75 years, each 20 mm Hg lower usual SBP was associated with approximately a halving in stroke mortality and a one-third reduction in IHD mortality and in all vascular mortality, with broadly similar associations observed in those with and without diabetes. Consequently, the absolute excess mortality associated with higher levels of blood pressure was substantially greater for those with diabetes, in particular those with poorly controlled diabetes. The single baseline measurement of SBP was more informative for vascular mortality than any of the other blood pressure indices considered. SBP was also log-linearly associated with kidney-disease mortality, both in individuals with and without a history of diabetes at recruitment.

The estimates of the shape and approximate strength of the associations of SBP with vascular mortality observed in the present study are consistent with previous reports from meta-analyses of cohort studies conducted in mostly high-income countries. For example, in the Prospective Studies Collaboration’s meta-analysis of data from 61 cohort studies involving 1 million individuals,^1^ each 20 mm Hg lower SBP was associated with more than a halving in stroke mortality between ages 40 and 69 years and approximately a halving in IHD mortality. The vascular mortality results in the present study are also consistent with more recent findings from large cohort studies in China^16^ and India.^17^ In contrast, there have been relatively few studies of blood pressure and risk of death in Hispanic populations, and the available studies have tended to be small and/or to have focused on hypertension as a dichotomous risk factor.^18,19,20^ However, a recent large study in Cuba reported that individuals with uncontrolled hypertension had about twice the cardiovascular mortality rate between ages 35 and 69 years compared with those without uncontrolled hypertension, which, given that there was a difference in mean SBP of about 20 mm Hg between these 2 groups, is also consistent with the results in this study.^20^ The observation that blood pressure was associated with kidney-related mortality in the present study is also consistent with a recent analysis of 0.5 million Chinese adults, in which 20 mm Hg lower SBP was associated with a halving in the incidence of CKD,^16^ although the causal relevance of these associations is uncertain given that CKD may be both a cause and consequence of hypertension.^21,22^

A key feature of the present study that distinguishes it from most previously studied populations is the high prevalence of obesity and uncontrolled diabetes in this Mexican population at recruitment.^7,23^ In the present analysis, those with previously diagnosed diabetes and poor glycemic control had more than 4 times the vascular mortality rate than those without diabetes. Consequently, the absolute relevance of blood pressure to vascular mortality was much greater in those with diabetes than in those without diabetes. Meta-analyses of randomized clinical trials have confirmed the benefits of lowering blood pressure both in those with and without diabetes^24^ as well as benefits of more intensive vs less intensive blood pressure lowering regimens.^25^ However, in the present study, only one-third of those with previously diagnosed diabetes were taking blood pressure–lowering medication at recruitment.^7^ Hence, the present study reinforces the need in Mexico for more widespread use of blood pressure–lowering medication and other inexpensive medications that lower vascular mortality (such as statins^26^), particularly among those with diabetes. The lack of any identifiable threshold level of blood pressure below which there was not a lower mortality risk also supports the most recent recommendations made by the European Society of Cardiology and European Society of Hypertension to reduce the treatment target level of blood pressure from below 140 mm Hg to below 130/80 mm Hg in most patients.^27^ From a global health perspective, the findings from this large previously understudied Hispanic population provide further support for the proposition that the association of blood pressure with cardiovascular disease risk is largely generalizable between different populations, a key assumption made by the Global Burden of Disease investigators when estimating the risks associated with elevated blood pressure and disability adjusted life-years lost worldwide.^4^

### Strengths and Limitations

The major strengths of the present study included the large population studied and prolonged duration of follow-up (yielding data on several thousands of deaths for analysis), the ability to exclude individuals with history of major chronic diseases, and the availability of information on a wide range of potential confounders. A further strength was the availability of repeat measurements of blood pressure in a sample of survivors at about 15 years after the baseline assessment, which allowed for an assessment of regression to the mean in blood pressure and appropriate correction for regression dilution bias.

This study did have limitations. By adopting a somewhat conservative approach, ie, estimating the regression dilution ratio at the midpoint of the follow-up period, we may have undercorrected for regression dilution bias. Previous studies have reported that most of the long-term regression to the mean occurs shortly after the initial measurement.^14^ The chief limitation of the current study was the lack of any information on nonfatal vascular events. In addition, the present study relied on causes of death listed on the death certificate. However, almost all deaths in Mexico are certified by a doctor and the overall accuracy and quality of certification of causes of death in Mexico is high.^28^ Moreover, the validity of the attributed causes of death on the death certificates is supported by the specificity of the associations identified; by contrast with the associations seen with vascular causes of death, SBP was unrelated to major nonvascular causes of death (including deaths from neoplastic and respiratory causes). Finally, although the 2 study districts were not representative of Mexico as a whole, prospective studies of nonrepresentative cohorts of individuals can provide reliable evidence about the associations of risk factors with disease that are widely generalizable.^29^

## Conclusions

Consistent with previous reports from high-income populations, the present study showed that blood pressure was strongly associated with both vascular and kidney-related mortality in this Mexican population with high levels of adiposity and diabetes. These findings suggest that medications to lower blood pressure should be used more widely in Mexico, especially among individuals with diabetes.

## Notes

Supplement. **eFigure 1.** Extent of Regression to the Mean in SBP and DBP Between Baseline and Resurvey, for Each Fifth of the Baseline Distribution **eFigure 2.** SBP, DBP and Use of Antihypertensive Drugs at Recruitment, by Age and Sex Among 141 074 Participants Without Prior Chronic Disease and Aged 35 to 84 Years at Recruitment **eFigure 3.** Association of SBP With Cause-Specific Vascular Mortality at Ages 75 to 84 Years **eFigure 4.** Association of SBP With Vascular Mortality by Age and Sex **eFigure 5.** Association of SBP With Vascular Mortality at Ages 35 to 74 Years by Levels of Confounders **eFigure 6.** Association of SBP With Vascular Mortality at Ages 35 to 74 Years Separately by Period of Follow-up **eFigure 7.** Association of SBP With Cause-Specific Nonvascular Mortality at Ages 35 to 74 Years **eFigure 8.** Association of SBP With Cause-Specific Nonvascular Mortality at Ages 35 to 74 Years, by History of Previously Diagnosed Diabetes **eTable 1.** Numbers of Deaths at Ages 35 to 74 Years by Underlying Cause (ICD-10 code) **eTable 2.** Baseline Characteristics of 7461 Participants Without Prior Chronic Disease and Aged 75 to 84 Years at Recruitment **eTable 3.** Baseline Characteristics of 9031 Resurveyed Participants Without Prior Chronic Disease and Aged 35 to 74 Years at Original Study Recruitment, by Sex **eTable 4.** Comparison of the Informativeness of Different Blood Pressure Indices (Measured Only Once) for Stroke, Ischaemic Heart Disease (IHD) and Vascular Mortality Rates at Ages 35 to 74 Years
