# Supplementary material for: Association of Blood Pressure With Cause-Specific Mortality in Mexican Adults
Source: JAMA Netw Open. 2020 Sep 25;3(9):e2018141. doi: 10.1001/jamanetworkopen.2020.18141 (PMC7519421; doi:10.1001/jamanetworkopen.2020.18141)
Supplement: Supplement. — eFigure 1. Extent of Regression to the Mean in SBP and DBP Between Baseline and Resurvey, for Each Fifth of the Baseline Distribution eFigure 2. SBP, DBP and Use of Antihypertensive Drugs at Recruitment, by Age and Sex Among 141 074 Participants Without Prior Chronic Disease and Aged 35 to 84 Years at Recruitment eFigure 3. Association of SBP With Cause-Specific Vascular Mortality at Ages 75 to 84 Years eFigure 4. Association of SBP With Vascular Mortality by Age and Sex eFigure 5. Association of SBP With Vascular Mortality at Ages 35 to 74 Years by Levels of Confounders eFigure 6. Association of SBP With Vascular Mortality at Ages 35 to 74 Years Separately by Period of Follow-up eFigure 7. Association of SBP With Cause-Specific Nonvascular Mortality at Ages 35 to 74 Years eFigure 8. Association of SBP With Cause-Specific Nonvascular Mortality at Ages 35 to 74 Years, by History of Previously Diagnosed Diabetes eTable 1. Numbers of Deaths at Ages 35 to 74 Years by Underlying Cause (ICD-10 code) eTable 2. Baseline Characteristics of 7461 Participants Without Prior Chronic Disease and Aged 75 to 84 Years at Recruitment eTable 3. Baseline Characteristics of 9031 Resurveyed Participants Without Prior Chronic Disease and Aged 35 to 74 Years at Original Study Recruitment, by Sex eTable 4. Comparison of the Informativeness of Different Blood Pressure Indices (Measured Only Once) for Stroke, Ischaemic Heart Disease (IHD) and Vascular Mortality Rates at Ages 35 to 74 Years [file jamanetwopen-e2018141-s001.pdf]

Supplementary Online Content

Tapia-Conyer R, Alegre-Díaz J, Gnatiuc L, et al. Association of blood pressure with cause-specific mortality in Mexican adults. *JAMA Netw Open*. 2020;3(9):e2018141. doi:10.1001/jamanetworkopen.2020.18141

eFigure 1. Extent of Regression to the Mean in SBP and DBP Between Baseline and Resurvey, for Each Fifth of the Baseline Distribution
eFigure 2. SBP, DBP and Use of Antihypertensive Drugs at Recruitment, by Age and Sex Among 141 074 Participants Without Prior Chronic Disease and Aged 35 to 84 Years at Recruitment
eFigure 3. Association of SBP With Cause-Specific Vascular Mortality at Ages 75 to 84 Years
eFigure 4. Association of SBP With Vascular Mortality by Age and Sex
eFigure 5. Association of SBP With Vascular Mortality at Ages 35 to 74 Years by Levels of Confounders
eFigure 6. Association of SBP With Vascular Mortality at Ages 35 to 74 Years Separately by Period of Follow-up
eFigure 7. Association of SBP With Cause-Specific Nonvascular Mortality at Ages 35 to 74 Years
eFigure 8. Association of SBP With Cause-Specific Nonvascular Mortality at Ages 35 to 74 Years, by History of Previously Diagnosed Diabetes
eTable 1. Numbers of Deaths at Ages 35 to 74 Years by Underlying Cause (*ICD-10* code)
eTable 2. Baseline Characteristics of 7461 Participants Without Prior Chronic Disease and Aged 75 to 84 Years at Recruitment
eTable 3. Baseline Characteristics of 9031 Resurveyed Participants Without Prior Chronic Disease and Aged 35 to 74 Years at Original Study Recruitment, by Sex
eTable 4. Comparison of the Informativeness of Different Blood Pressure Indices (Measured Only Once) for Stroke, Ischaemic Heart Disease (IHD) and Vascular Mortality Rates at Ages 35 to 74 Years

This supplementary material has been provided by the authors to give readers additional information about their work.

© 2020 Tapia-Conyer R et al. *JAMA Network Open.*

eFigure 1: Extent of Regression to the Mean in SBP and DBP Between Baseline and Resurvey, for each Fifth of the Baseline Distribution

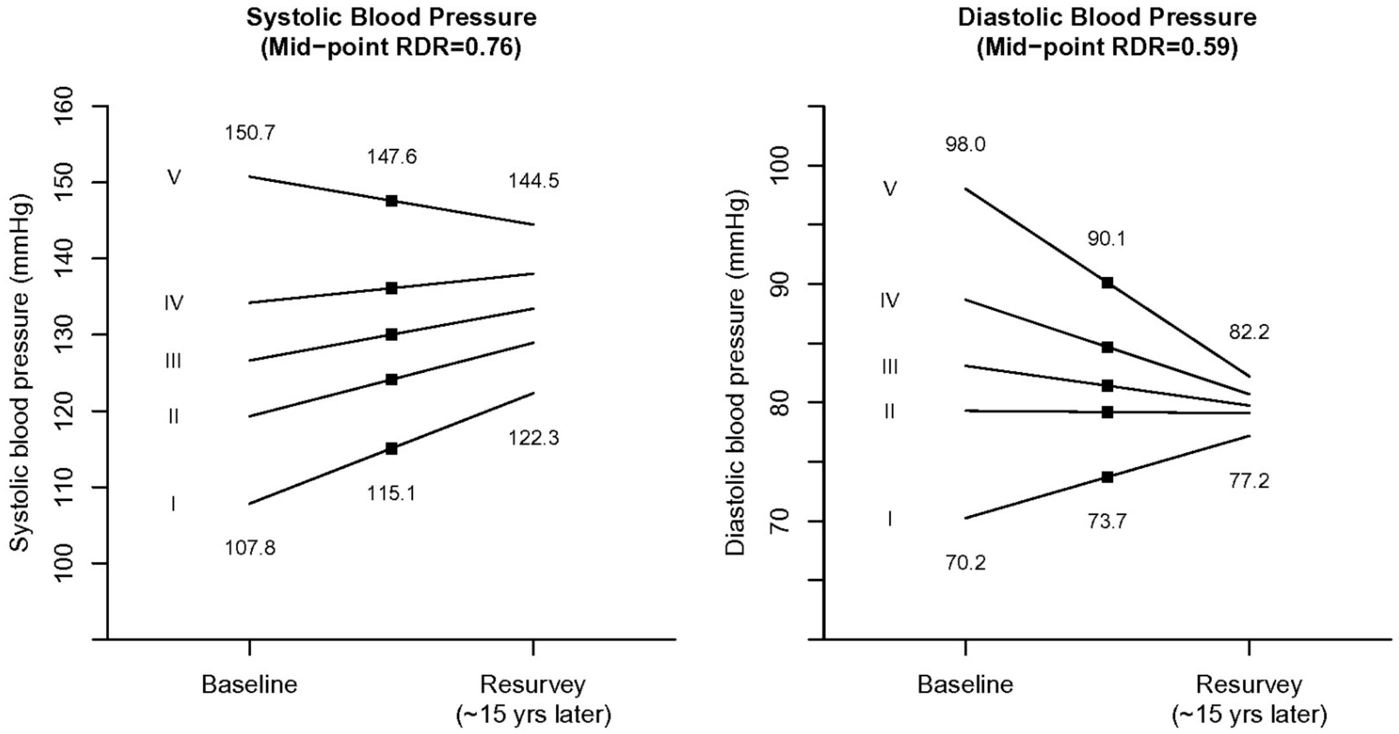

Analysis restricted to participants aged 35−74 and without prior chronic disease (chronic kidney disease, ischemic heart disease, stroke, cirrhosis, cancer, or emphysema) at recruitment, with complete data on analysis covariates (see Table 1) and with complete, plausible, blood pressure data at both recruitment and resurvey. The regression dilution ratio (RDR) is calculated as the ratio of the range of mid−point to baseline means; for example for SBP this equates to (147.6−115.1)/(150.7−107.8)=0.76.

© 2020 Tapia-Conyer R et al. *JAMA Network Open.*

eFigure 2: SBP, DBP and Use of Antihypertensive Drugs at Recruitment, by Age and Sex Among 141 074 Participants Without Prior Chronic Disease and Aged 35 to 84 Years at Recruitment

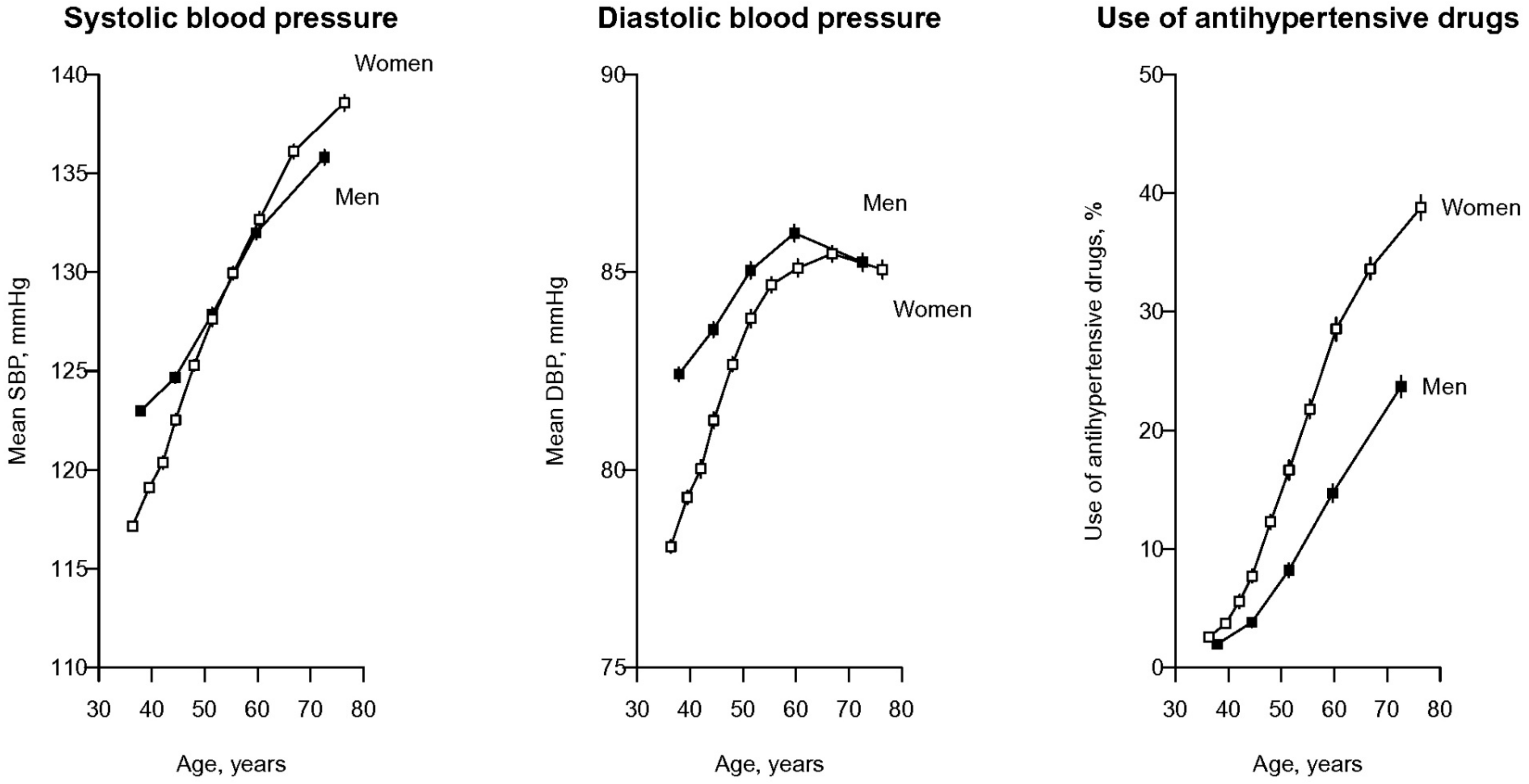

Results shown for five equally-sized groups of men and ten equally-sized groups of women (just under 10 000 participants in each group). Vertical lines representing 95% confidence intervals are drawn, but are mostly contained within the height of each plotting symbol.

© 2020 Tapia-Conyer R et al. *JAMA Network Open.*

eFigure 3: Association of SBP with Cause−Specific Vascular Mortality at Ages 75 to 84 Years

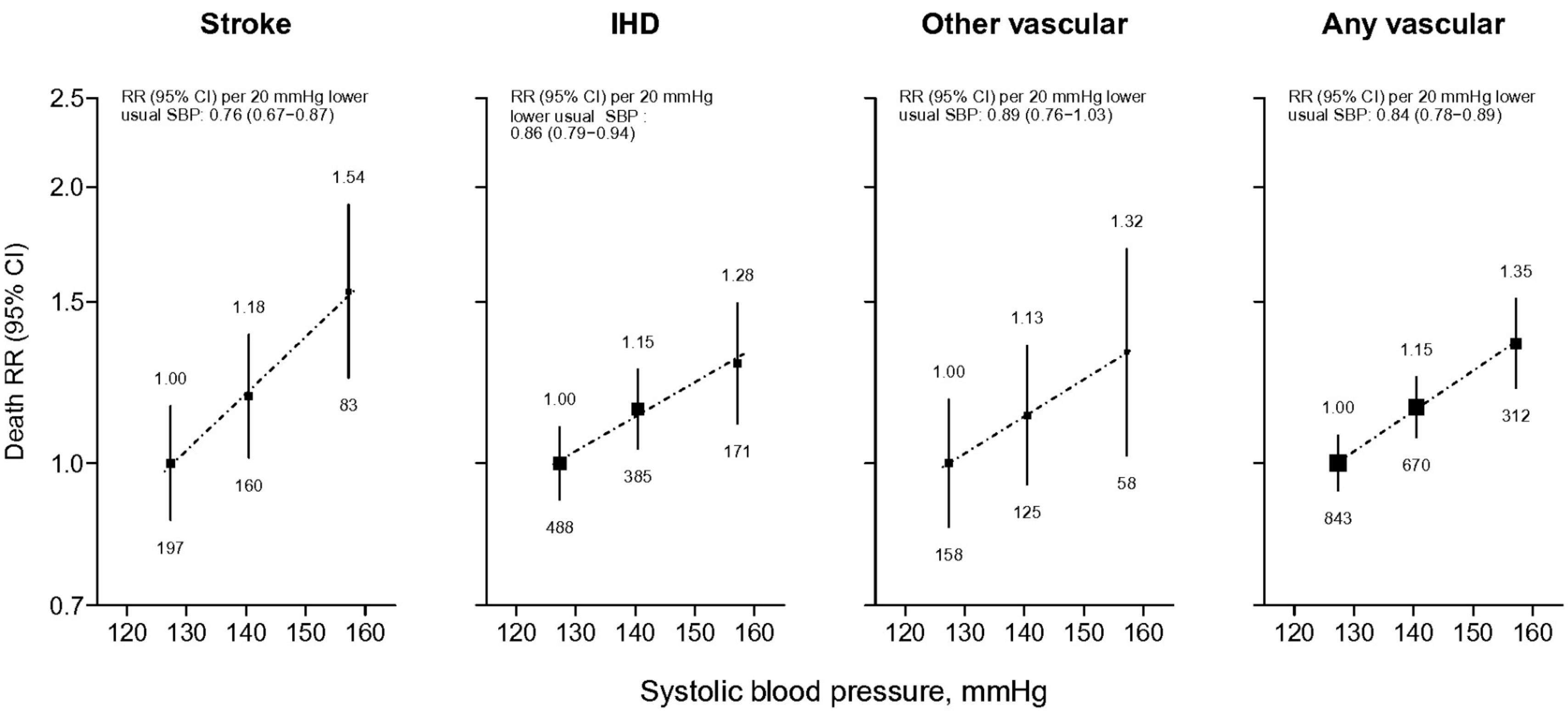

Analyses and exclusions as for Figure 1, except that analyses are for ages 75−84. Due to smaller numbers, the first two, middle two and last two SBP categories have each been combined to give three groups.

© 2020 Tapia-Conyer R et al. *JAMA Network Open.*

eFigure 4: Association of SBP With Vascular Mortality by Age and Sex

Age (years)	Sex	No. of deaths	RR (95% CI) per 20 mmHg lower usual SBP	p−value
35−54	Men	177	0.42 (0.34−0.54)	<0.001
	Women	187	0.58 (0.46−0.74)	<0.001
	**Both**	**364**	**0.50 (0.43−0.59)**	**<0.001**
55−64	Men	356	0.57 (0.49−0.67)	<0.001
	Women	420	0.57 (0.50−0.65)	<0.001
	**Both**	**776**	**0.57 (0.51−0.63)**	**<0.001**
65−74	Men	627	0.70 (0.63−0.79)	<0.001
	Women	741	0.73 (0.66−0.80)	<0.001
	**Both**	**1368**	**0.72 (0.67−0.77)**	**<0.001**

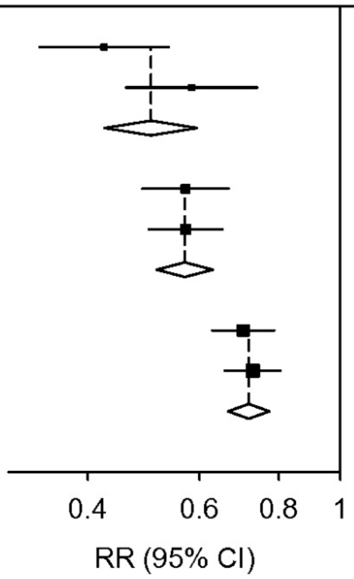

Analyses and exclusions as for Figure 2, except that RRs are instead estimated separately in men and women (and adjusted for diabetes status). Within each age−group shown, the RRs shown are consistent in men and women (all three tests for heterogeneity give p>0.05).

© 2020 Tapia-Conyer R et al. *JAMA Network Open.*

eFigure 5: Association of SBP with Vascular Mortality at Ages 35 to 74 Years by Levels of Confounders

Variable	Number of deaths	Vascular mortality RR (95% CI) per 20 mmHg lower SBP	Heterogeneity/ trend
**Age at risk (years)**			
35–54	364	0.50 (0.43–0.60)	$\chi^2_1 = 21.3$
55–64	776	0.57 (0.51–0.63)	(p<0.001)
65–74	1368	0.72 (0.67–0.77)	
**Sex**			
Male	1160	0.63 (0.58–0.68)	$\chi^2_1 = 0.7$
Female	1348	0.66 (0.61–0.71)	(p=0.39)
**District**			
Coyoácan	877	0.68 (0.62–0.74)	$\chi^2_1 = 1.7$
Iztapalapa	1631	0.63 (0.58–0.67)	(p=0.20)
**Smoking**			
Never	1082	0.69 (0.63–0.75)	$\chi^2_2 = 4.8$
Former	558	0.61 (0.54–0.68)	(p=0.09)
Current	868	0.61 (0.55–0.67)	
**Education**			
University	244	0.53 (0.44–0.65)	$\chi^2_1 = 13.1$
High school	481	0.57 (0.50–0.66)	(p<0.001)
Elementary	1429	0.65 (0.60–0.70)	
Other	354	0.79 (0.68–0.92)	
**Waist circumference**			
I (mean: 81cm)	430	0.70 (0.61–0.81)	$\chi^2_1 = 0.3$
II	565	0.62 (0.55–0.69)	(p=0.58)
III	654	0.65 (0.58–0.73)	
IV (mean: 109cm)	859	0.64 (0.58–0.71)	
**Hip circumference**			
I (mean: 93cm)	728	0.69 (0.62–0.77)	$\chi^2_1 = 0.9$
II	581	0.62 (0.55–0.69)	(p=0.35)
III	554	0.61 (0.55–0.69)	
IV (mean: 119cm)	645	0.64 (0.58–0.72)	
**Weight**			
I (mean: 56kg)	611	0.72 (0.65–0.81)	$\chi^2_1 = 0.4$
II	637	0.58 (0.52–0.65)	(p=0.54)
III	548	0.62 (0.55–0.70)	
IV (mean: 88kg)	712	0.67 (0.60–0.75)	
**Height**			
I (mean: 146cm)	645	0.67 (0.61–0.75)	$\chi^2_1 = 0.04$
II	561	0.62 (0.55–0.70)	(p=0.84)
III	604	0.60 (0.53–0.67)	
IV (mean: 168cm)	698	0.68 (0.60–0.76)	
**Physical activity**			
None	2005	0.65 (0.61–0.69)	$\chi^2_1 = 0.1$
Up to twice weekly	174	0.59 (0.47–0.75)	(p=0.70)
At least three times weekly	329	0.64 (0.54–0.75)	
**Alcohol consumption**			
None	500	0.71 (0.63–0.81)	$\chi^2_2 = 3.3$
Former	126	0.60 (0.46–0.79)	(p=0.19)
Current	1882	0.63 (0.59–0.67)	
**Diabetes status**			
No diabetes	1267	0.59 (0.55–0.64)	$\chi^2_1 = 3.0$
Undiagnosed diabetes	226	0.73 (0.60–0.89)	(p=0.08)
Dx diabetes, HbA1c<9%	393	0.73 (0.64–0.83)	
Dx diabetes, $9 \leq$ HbA1c<11%	287	0.70 (0.60–0.83)	
Dx diabetes, HbA1c $\geq$ 11%	335	0.63 (0.54–0.74)	
**Overall**	**2508**	**0.65 (0.61–0.68)**	

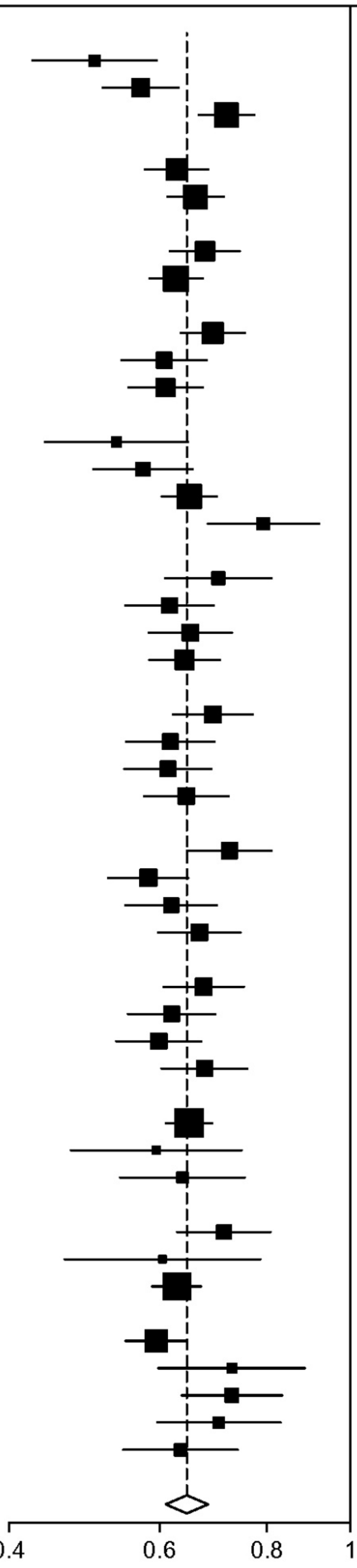

Exclusions and analyses as for Figure 2, with the exception that RRs are now estimated separately by levels of each confounder shown (with mutual adjustment for the other shown confounders).

© 2020 Tapia-Conyer R et al. *JAMA Network Open.*

eFigure 6: Association of SBP With Vascular Mortality at Ages 35 to 74 Years Separately by Period of Follow−up

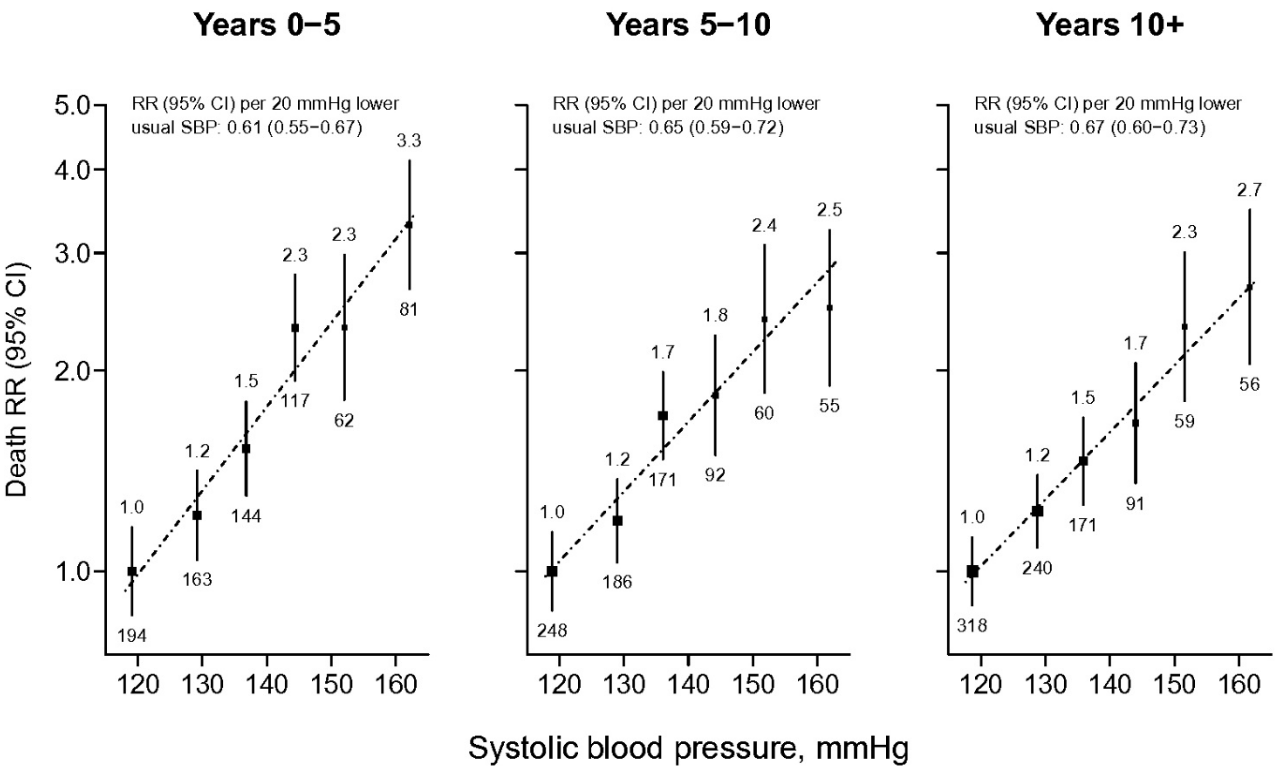

Analyses and exclusions as for Figure 1, except that analyses are now done separately by period of follow−up.

© 2020 Tapia-Conyer R et al. *JAMA Network Open.*

eFigure 7: Association of SBP with Cause−Specific Nonvascular Mortality at Ages 35 to 74 Years

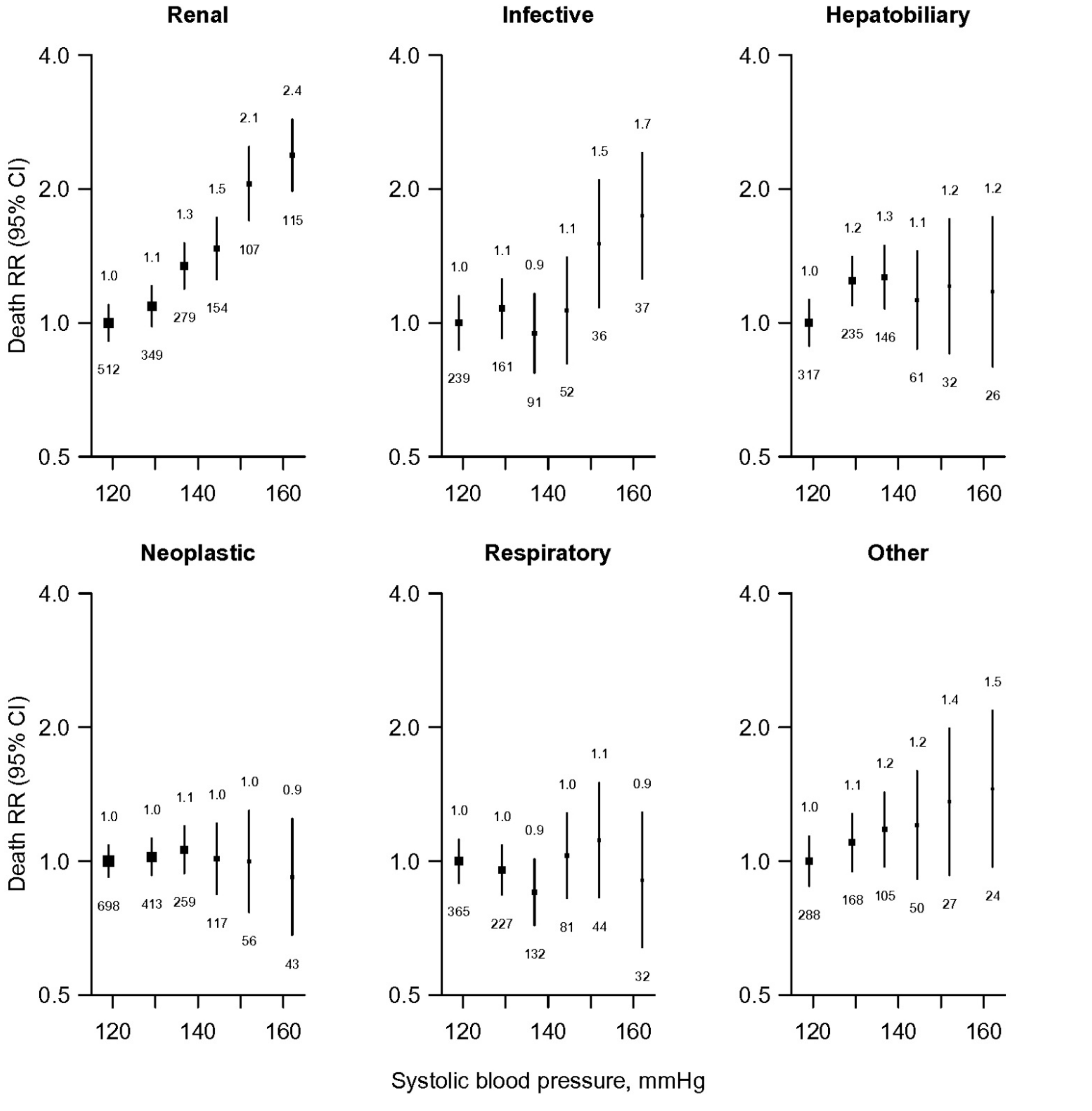

Analyses and exclusions as for Figure 1. Infective endpoint excludes respiratory infections (which are included in respiratory).

© 2020 Tapia-Conyer R et al. *JAMA Network Open.*

eFigure 8: Association of SBP with Cause−Specific Nonvascular Mortality at Ages 35 to 74 Years, by History of Previously Diagnosed Diabetes

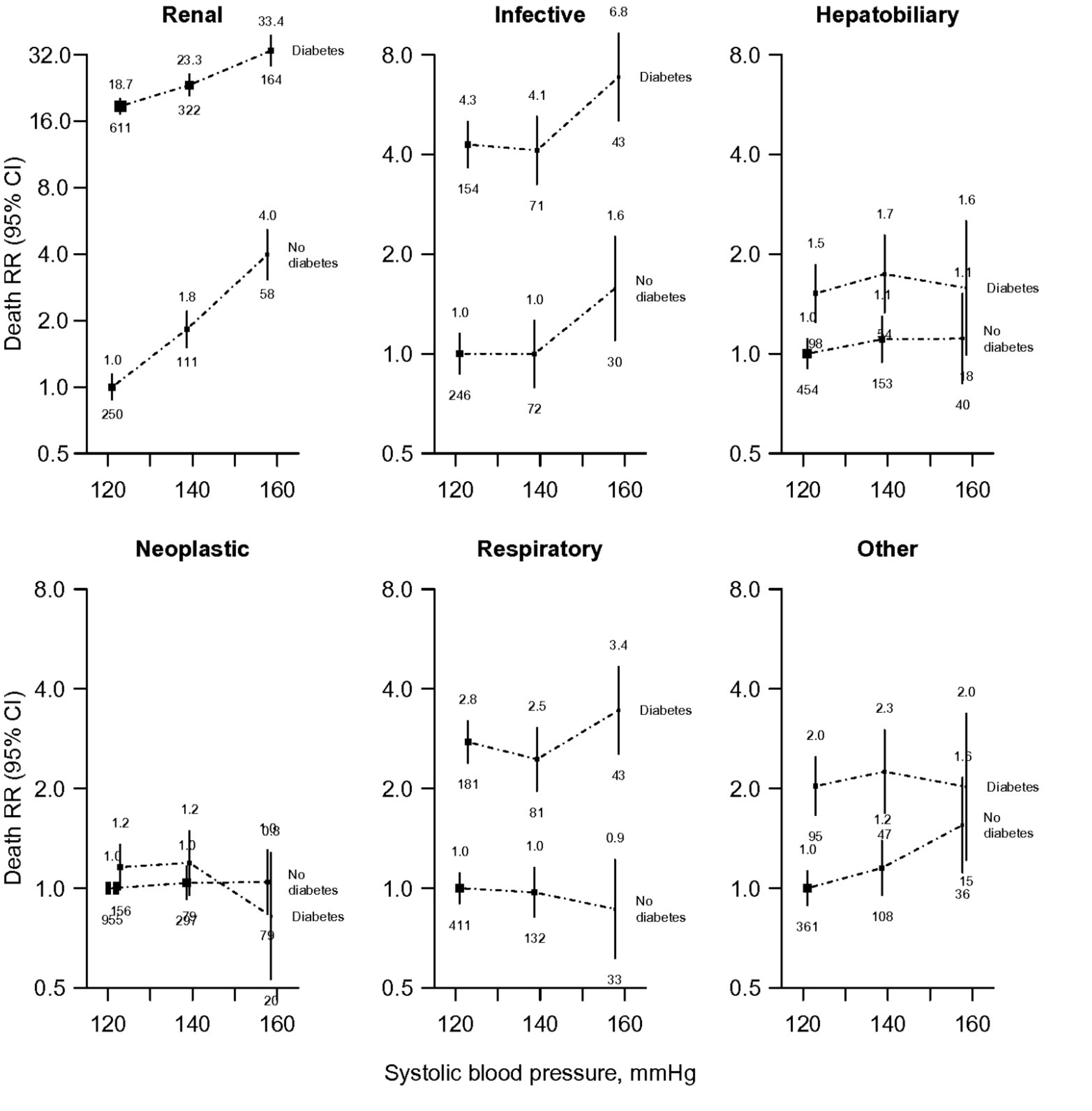

Analyses and exclusions as for Figure 1, but now separately by, instead of adjusted for, diabetes. Infective endpoint excludes respiratory infections (which are included in respiratory).

© 2020 Tapia-Conyer R et al. *JAMA Network Open.*

eTable 1: Numbers of Deaths at Ages 35 to 74 Years by Underlying Cause (ICD-10 code)

Underlying cause of death	ICD-10 codes (and number of deaths)
Stroke (n=574)	I600 (1), I602 (1), I608 (1), I609 (57), I61 (1), I610 (1), I612 (1), I614 (1), I615 (2), I618 (1), I619 (178), I620 (7), I629 (3), I633 (2), I634 (11), I635 (2), I638 (2), I639 (44), I649 (1), I64X (77), I669 (5), I671 (6), I672 (1), I673 (1), I674 (2), I678 (56), I679 (86), I693 (4), I694 (3), I698 (16)
IHD (n=1406)	I200 (4), I209 (2), I210 (16), I211 (8), I219 (1270), I221 (1), I249 (7), I251 (30), I252 (1), I255 (1), I258 (6), I259 (60)
Other vascular (n=528)	E115 (25), E145 (25), I050 (1), I051 (1), I059 (13), I070 (1), I071 (2), I080 (2), I081 (1), I091 (1), I099 (13), I110 (72), I119 (8), I260 (2), I269 (53), I270 (6), I272 (2), I279 (6), I301 (1), I319 (1), I330 (5), I340 (3), I348 (1), I350 (11), I351 (1), I358 (1), I38X (5), I420 (7), I429 (1), I442 (5), I443 (1), I460 (1), I469 (8), I471 (1), I472 (2), I489 (2), I48X (5), I490 (4), I500 (35), I501 (10), I509 (47), I515 (1), I518 (2), I519 (10), I710 (5), I712 (1), I713 (4), I714 (1), I718 (3), I719 (1), I729 (1), I739 (2), I741 (1), I771 (11), I776 (1), I802 (6), I803 (1), I822 (1), I828 (1), I829 (3), I830 (1), I872 (4), I879 (1), I99X (2), K550 (52), K551 (1), K552 (1), K559 (5), K761 (1), Q210 (1), Q231 (1), Q238 (1), Q248 (1), R570 (8)
Renal (n=1516)	E102 (6), E112 (655), E122 (1), E142 (285), I120 (79), I129 (2), I130 (1), I131 (1), I132 (21), N002 (1), N039 (23), N049 (1), N059 (3), N10X (2), N12X (7), N142 (1), N151 (9), N170 (1), N179 (64), N180 (9), N185 (17), N189 (135), N19X (34), N200 (6), N201 (1), N289 (1), N390 (148), Q619 (1), Y841 (1)
Infective (n=616)	A047 (2), A060 (1), A090 (12), A099 (28), A09X (10), A170 (1), A180 (1), A181 (1), A182 (1), A183 (1), A188 (1), A199 (3), A415 (1), A419 (155), A483 (1), A498 (1), A810 (2), A86X (2), B200 (1), B201 (1), B207 (2), B208 (6), B210 (1), B212 (1), B227 (1), B238 (4), B24X (5), B690 (2), B948 (1), G009 (3), G039 (4), G049 (6), G060 (1), H440 (1), K052 (1), K222 (1), K223 (2), K228 (1), K251 (3), K254 (8), K255 (7), K256 (2), K259 (5), K264 (5), K265 (2), K269 (1), K274 (3), K275 (1), K290 (5), K291 (2), K292 (1), K295 (3), K297 (1), K318 (6), K352 (4), K353 (3), K358 (4), K359 (1), K572 (2), K578 (4), K579 (9), K610 (2), K611 (1), K650 (9), K658 (1), K659 (47), K920 (8), K921 (1), K922 (95), L021 (3), L022 (6), L023 (1), L024 (1), L031 (3), L038 (1), L039 (3), L089 (18), L899 (3), L89X (4), L905 (1), L984 (5), M009 (1), M600 (2), M725 (1), M726 (12), M798 (22), M869 (2), N410 (1), N498 (6), N499 (1), N719 (1), N739 (2), N764 (1)
Hepatobiliary (n=817)	B169 (2), B171 (21), B181 (2), B182 (8), B189 (1), B190 (2), I850 (12), I859 (4), K563 (1), K701 (18), K702 (1), K703 (131), K704 (13), K709 (12), K711 (2), K716 (1), K720 (6), K721 (39), K729 (139), K739 (2), K742 (1), K743 (3), K745 (1), K746 (221), K750 (9), K754 (2), K759 (1), K766 (10), K767 (10), K769 (15), K800 (4), K801 (7), K802 (2), K803 (4), K804 (1), K805 (3), K810 (15), K811 (3), K819 (4), K822 (1), K829 (5), K830 (14), K831 (2), K852 (4), K858 (7), K859 (22), K85X (23), K861 (2), K868 (3), Q447 (1)
Neoplastic (n=1586)	C029 (7), C049 (1), C069 (1), C07X (1), C089 (1), C109 (3), C119 (1), C139 (1), C140 (3), C159 (11), C160 (2), C169 (146), C170 (6), C179 (1), C182 (1), C184 (1), C187 (2), C189 (89), C19X (4), C20X (15), C220 (38), C221 (14), C229 (82), C23X (20), C240 (7), C241 (8), C248 (3), C249 (14), C250 (19), C259 (71), C260 (2), C269 (2), C319 (1), C329 (12), C33X (1), C349 (126), C37X (1), C383 (1), C384 (1), C412 (2), C414 (1), C419 (6), C437 (1), C438 (1), C439 (12), C445 (1), C447 (1), C449 (5), C450 (1), C451 (1), C459 (3), C469 (1), C479 (1), C480 (9), C482 (5), C492 (3), C499 (7), C509 (147), C519 (2), C539 (87), C541 (13), C549 (2), C55X (10), C56X (78), C609 (1), C61X (48), C629 (1), C64X (60), C679 (18), C680 (2), C709 (2), C710 (13), C711 (1), C718 (1), C719 (28), C729 (1), C73X (19), C741 (1), C749 (1), C751 (1), C759 (1), C760 (4), C763 (2), C764 (1), C765 (1), C780 (4), C786 (1), C787 (8), C788 (2), C793 (2), C794 (3), C795 (1), C796 (1), C798 (2), C800 (23), C809 (11), C80X (1), C817 (1), C819 (8), C829 (1), C833 (10), C838 (1), C839 (2), C844 (1), C845 (1), C851 (1), C857 (1), C859 (37), C900 (29), C901 (1), C902 (1), C910 (21), C911 (1), C920 (25), C921 (6), C927 (2), C929 (2), C950 (1), C959 (3), C97X (1), D371 (2), D372 (1), D374 (3), D376 (6), D377 (1), D380 (2), D381 (4), D383 (2), D391 (2), D410 (2), D419 (1), D430 (7), D432 (1), D449 (1), D486 (1), D487 (5), D489 (2)
Respiratory* (n=881)	A162 (8), A165 (1), A169 (2), B206 (2), B441 (1), B909 (1), E848 (1), J069 (1), J09 (3), J100 (2), J111 (1), J151 (3), J157 (1), J159 (21), J180 (48), J181 (25), J182 (1), J188 (1), J189 (380), J209 (3), J22X (16), J348 (1), J391 (1), J42X (12), J439 (20), J440 (71), J448 (4), J449 (112), J459 (9), J46X (2), J47X (2), J60X (1), J64X (5), J677 (1), J679 (2), J680 (1), J690 (1), J80X (1), J81X (3), J841 (65), J849 (6), J850 (1), J852 (1), J869 (5), J90X (3), J939 (1), J960 (2), J961 (1), J969 (1), J980 (1), J981 (1), J984 (10), J985 (4), J988 (7), Q311 (1)

IHD=Ischemic heart disease. *Includes respiratory infections.

eTable 2: Baseline Characteristics of 7461 Participants Without Prior Chronic Disease and Aged 75 to 84 Years at Recruitment

	Baseline systolic blood pressure (participants)						
	<125 mmHg (n=1732)	125-134 mmHg (n=1792)	135-144 mmHg (n=1636)	145-154 mmHg (n=1075)	155-164 mmHg (n=632)	≥165 mmHg (n=594)	Overall (n=7461)
Age, years	79 (3)	79 (3)	79 (3)	79 (3)	79 (3)	79 (3)	79 (3)
Male	748 (43%)	680 (38%)	594 (36%)	382 (36%)	197 (31%)	188 (32%)	2789 (37%)
**Blood pressure**							
Systolic blood pressure, mmHg	116 (8)	130 (3)	140 (3)	150 (3)	159 (3)	179 (14)	138 (19)
Diastolic blood pressure, mmHg	75 (8)	83 (7)	87 (8)	89 (9)	92 (10)	97 (13)	85 (11)
Taking blood pressure lowering medication	344 (20%)	538 (30%)	593 (36%)	453 (42%)	296 (47%)	337 (57%)	2561 (34%)
**Socio-economic status and lifestyle behaviours**							
Resident of Coyoacán	480 (28%)	592 (33%)	586 (36%)	381 (35%)	218 (34%)	264 (44%)	2521 (34%)
University/college educated	46 (3%)	72 (4%)	70 (4%)	36 (3%)	16 (3%)	21 (4%)	261 (3%)
Current smoker	259 (15%)	249 (14%)	226 (14%)	149 (14%)	86 (14%)	65 (11%)	1034 (14%)
Current drinker	1203 (69%)	1254 (70%)	1106 (68%)	729 (68%)	432 (68%)	395 (66%)	5119 (69%)
Any regular leisure-time physical activity	349 (20%)	357 (20%)	301 (18%)	218 (20%)	114 (18%)	132 (22%)	1471 (20%)
**Physical measurements**							
BMI, kg/m²	26.7 (4.4)	27.6 (4.5)	27.9 (4.7)	28.0 (4.6)	28.2 (4.6)	28.1 (4.7)	27.6 (4.6)
Waist circumference, cm	95 (11)	97 (11)	97 (11)	98 (11)	97 (11)	97 (11)	97 (11)
Hip circumference, cm	101 (10)	103 (11)	104 (10)	104 (11)	104 (10)	104 (10)	103 (10)
**Diabetes and glycosylated hemoglobin**							
Diabetes status							
No diabetes	1298 (75%)	1284 (72%)	1171 (72%)	749 (70%)	462 (73%)	410 (69%)	5374 (72%)
Undiagnosed diabetes	102 (6%)	115 (6%)	119 (7%)	88 (8%)	39 (6%)	32 (5%)	495 (7%)
Diagnosed diabetes with HbA1c <9%	225 (13%)	272 (15%)	243 (15%)	179 (17%)	84 (13%)	100 (17%)	1103 (15%)
Diagnosed diabetes with 9 ≤ HbA1c <11%	69 (4%)	80 (4%)	59 (4%)	41 (4%)	38 (6%)	31 (5%)	318 (4%)
Diagnosed diabetes with HbA1c ≥ 11%	38 (2%)	41 (2%)	44 (3%)	18 (2%)	9 (1%)	21 (4%)	171 (2%)
**Medication use**							
Any anti-thrombotic	90 (5%)	88 (5%)	77 (5%)	53 (5%)	37 (6%)	39 (7%)	384 (5%)
Any lipid lowering	11 (1%)	9 (1%)	11 (1%)	7 (1%)	5 (1%)	2 (<0.5%)	45 (1%)

Mean (SD) or n (column %) shown. BMI=Body mass index. Table excludes those with prior chronic disease at recruitment (ischemic heart disease, stroke, chronic kidney disease, cirrhosis, cancer, emphysema), missing or out-of-range data on any analysis covariate (sex, district of residence, educational level attained, anthropometry, smoking status, alcohol intake, leisure time physical activity, diabetes status), uncertain follow-up, or missing or out-of-range blood pressure (defined as SBP <80 or >250 mmHg, DBP <40 or >150 mmHg, or pulse pressure <15 mmHg).

© 2020 Tapia-Conyer R et al. *JAMA Network Open.*

eTable 3: Baseline Characteristics of 9031 Resurveyed Participants Without Prior Chronic Disease and Aged 35 to 74 Years at Original Study Recruitment, by Sex

	Men (n=2705)	Women (n=6326)	All (n=9031)
Age, years	52 (10)	50 (10)	51 (10)
**Blood pressure**			
Systolic blood pressure, mmHg	128 (15)	127 (16)	127 (16)
Diastolic blood pressure, mmHg	85 (10)	83 (10)	83 (10)
Taking blood pressure lowering medication	219 (8%)	917 (14%)	1136 (13%)
**Socio-economic status and lifestyle behaviours**			
Resident of Coyoacán	1536 (57%)	3285 (52%)	4821 (53%)
University/college educated	516 (19%)	571 (9%)	1087 (12%)
Current smoker	1420 (52%)	1291 (20%)	2711 (30%)
Current drinker	2336 (86%)	4422 (70%)	6758 (75%)
Any regular leisure-time physical activity	813 (30%)	1083 (17%)	1896 (21%)
**Physical measurements**			
BMI, kg/m²	28.2 (4.1)	29.9 (5.1)	29.4 (4.9)
Waist circumference, cm	97 (10)	94 (12)	94 (11)
Hip circumference, cm	101 (7)	107 (11)	105 (10)
**Diabetes and glycosylated hemoglobin**			
Diabetes status			
No diabetes	2307 (85%)	5411 (86%)	7718 (85%)
Undiagnosed diabetes	143 (5%)	321 (5%)	464 (5%)
Diagnosed diabetes with HbA1c <9%	148 (5%)	332 (5%)	480 (5%)
Diagnosed diabetes with 9 ≤ HbA1c <11%	71 (3%)	145 (2%)	216 (2%)
Diagnosed diabetes with HbA1c ≥ 11%	36 (1%)	117 (2%)	153 (2%)
**Medication use**			
Any anti-thrombotic	42 (2%)	146 (2%)	188 (2%)
Any lipid lowering	12 (<0.5%)	27 (<0.5%)	39 (<0.5%)

Mean (SD) or n (column %) shown. BMI=Body mass index. Table excludes those with prior chronic disease at recruitment (ischemic heart disease, stroke, chronic kidney disease, cirrhosis, cancer, emphysema), missing or out-of-range baseline data on any analysis covariate (sex, district of residence, educational level attained, anthropometry, smoking status, alcohol intake, leisure time physical activity, diabetes status), uncertain follow-up, or missing or out-of-range blood pressure (at baseline or resurvey).

© 2020 Tapia-Conyer R et al. *JAMA Network Open.*

eTable 4: Comparison of the Informativeness of Different Blood Pressure Indices (Measured Only Once) for Stroke, Ischemic Heart Disease (IHD) and Vascular Mortality Rates at Ages 35 to 74 Years

	Relative informativeness* for		
**Blood pressure index**	**Stroke mortality**	**IHD mortality**	**Vascular mortality**
Systolic blood pressure (SBP)	100%	100%	100%
Diastolic blood pressure (DBP)	69%	52%	62%
Mid blood pressure (1/2 SBP + 1/2 DBP)	98%	90%	95%
Mean arterial pressure (2/3 DBP + 1/3 SBP)	93%	81%	89%
Pulse pressure (SBP - DBP)	54%	69%	60%

IHD=Ischemic heart disease. *Informativeness of the given index (as indicated by the age-stratified and confounder-adjusted χ^2 statistic relating it to cause-specific mortality), as a percentage of the informativeness of the systolic blood pressure. Estimates of the informativeness of each blood pressure index are adjusted for age, sex, location, education alcohol intake, leisure-time physical activity, anthropometry and diabetes status.

© 2020 Tapia-Conyer R et al. *JAMA Network Open.*
